# Permanent and Transient Congenital Hypothyroidism in Fayoum, Egypt: A Descriptive Retrospective Study

**DOI:** 10.1371/journal.pone.0068048

**Published:** 2013-06-28

**Authors:** Osama E. M. Bekhit, Remon M. Yousef

**Affiliations:** 1 Pediatrics department, Fayoum Faculty of Medicine, Fayoum University, Egypt

## Abstract

**Background:**

Congenital hypothyroidism (CH) is one of the most common preventable causes of mental retardation. One important challenge in understanding the epidemiology of CH is that some newborns will have transient CH, a temporary depression of thyroid hormone concentrations that can last from several days to several months. Studies from other countries have reported that 10 to 15% of children treated for CH ultimately prove not to need treatment past 3 years of age to maintain normal hormone concentrations, and thus have transient hypothyroidism. The purpose of this study was to determine the prevalence of permanent and transient congenital hypothyroidism in Fayoum, Egypt.

**Methods:**

Cases detected by Fayoum neonatal screening program (NSP) between January 2003 and December 2011, and followed up at health insurance center were included. Permanent or transient CH was determined using results of thyroid function tests.

**Results:**

Of the 248 patients diagnosed primarily with CH by NSP; 204 (82.3%) patients were diagnosed to have permanent CH (prevalence 1/3587 live birth), and 44 (17.7%) patients were diagnosed to have transient CH (prevalence 1/16667 live birth). Initial TSH levels were higher in permanent CH cases than transient cases (p<0.004). Female to male ratio was 0.8 and 0.7 in permanent and transient CH respectively. 161 (65%) patients had thyroid dysgenesis (107 ectopic thyroid gland, 28 athyreosis and 26 thyroid hypoplasia). 87 (35%) patients had intact gland in thyroid scan and were considered to have dyshormonogenesis. Of these 87 patients 44 proved to have transient CH and 43 had permanent CH.

**Conclusion:**

The preliminary data from our study revealed that the incidences of CH as well as the permanent form were similar to worldwide reports. Although the high incidence of transient CH in our study could be explained by iodine deficiency further studies are needed to confirm the etiology and plan the treatment strategies.

## Introduction

Congenital hypothyroidism (CH) is one of the most common preventable causes of mental retardation. Neonatal screening programs allow for the early detection and treatment of CH, thus preventing the mental retardation that results from the lack of thyroid hormone [1].

Despite the unquestioned public health success of newborn screening programs and management of CH, there are still gaps in knowledge. For example, one important challenge in understanding the epidemiology of CH is that some newborns will have transient CH, a temporary depression of thyroid hormone concentrations that can last from several days to several months [2].

**Figure 1:**
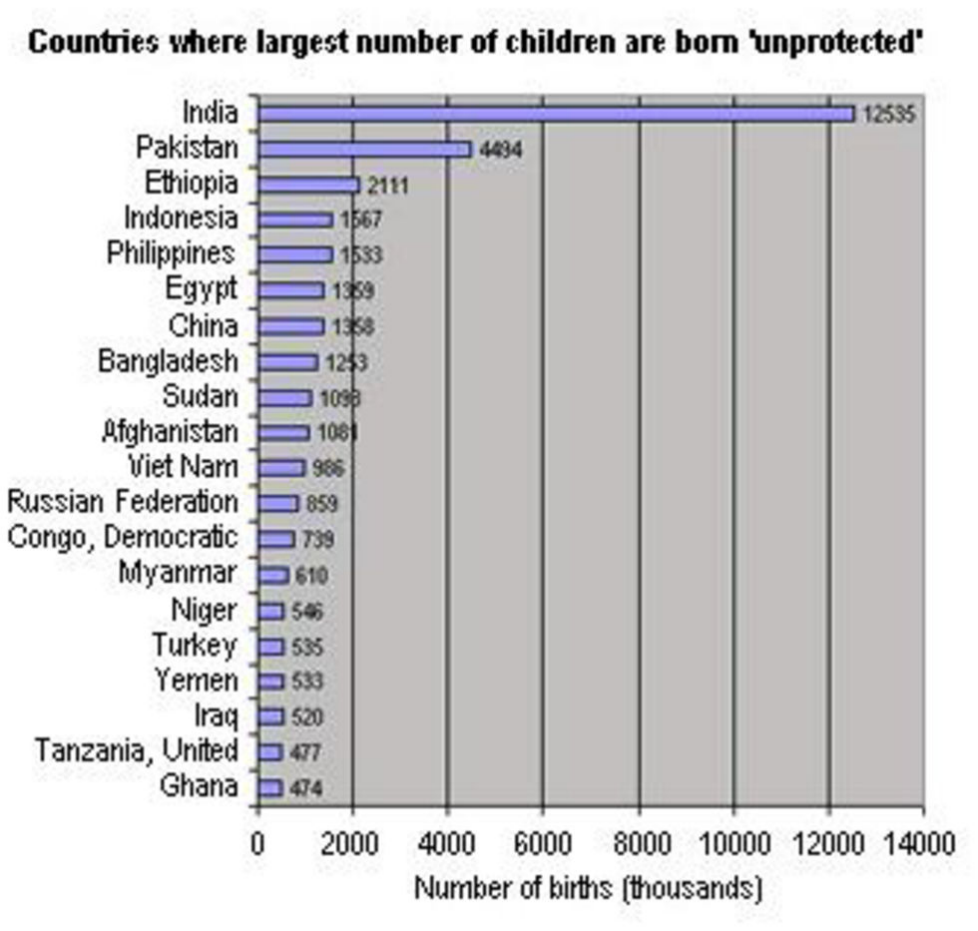
Countries where largest number of children are born unprotected [11].

**Figure 2:**
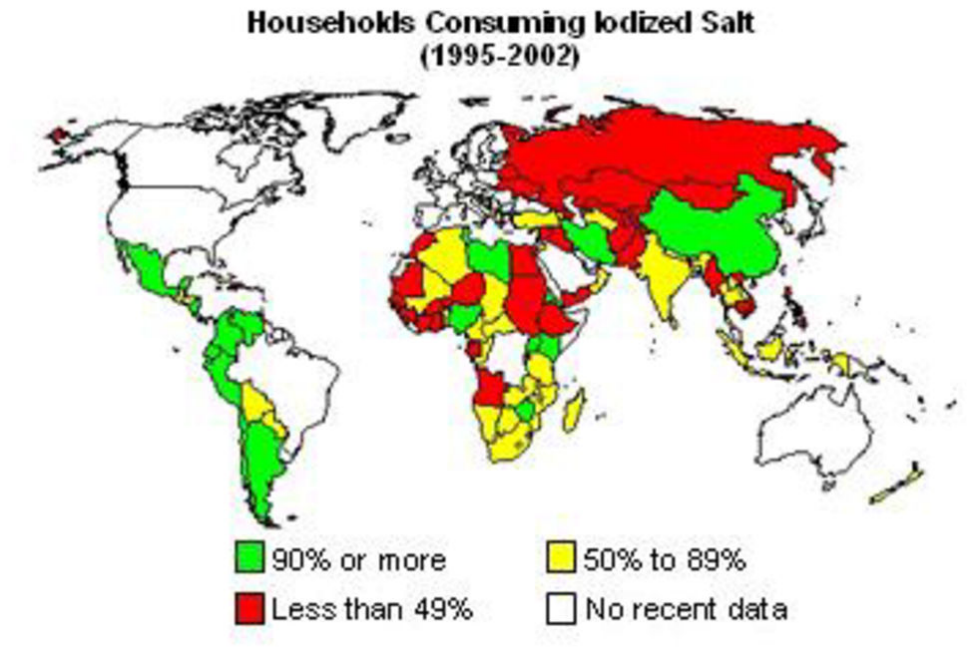
Households consuming iodized salt (1995–2002)[11].

Transient congenital hypothyroidism reverts later to normal, which may or may not require replacement therapy. Its incidence varies depending on whether the condition is defined on the basis of abnormal neonatal screening tests for congenital hypothyroidism alone or whether the diagnosis is considered only if the abnormality persists in the confirmatory tests [3]. Permanent dysfunction mainly results from mal-development, absence or ectopic thyroid gland, whereas the underlying causes of transient functional impairment are less clear and may include maternal factors such as iodine deficiency, excessive iodine intake, anti-thyroid medication or presence of antibodies against thyroid tissue during pregnancy [3,4]. Also neonatal very low birth weight (<1500gm) and prematurity (<37 weeks gestation), immaturity of thyroidal iodine organification, exposure to excess iodine (e.g. use of iodinated disinfectants or contrast agents) and gene mutation may contribute to transient CH [5–7,8]. Diagnosis of transient hypothyroidism is important to avoid lifelong unnecessary therapy with its possible side effects [9]. Besides, the governmental financial burden for this unnecessary therapy could be invested in other purposive health services. Iodine deficiency is a global health problem. Goiter has been known to exist in Egypt since Ancient times, Papyrus dating since 1500 BC reported thyroidectomy and there are suggestions that Cleopatra had goiter [10]. The UNICEF categorized Egypt as one of the countries with low iodinated salt consumption with large number of neonates exposed to iodine deficiency (figures 1 & 2) [11]. Moreover our governorate, being an oasis is one of the areas that are highly affected by iodine deficiency in soil and food sources. With this background this descriptive retrospective study, analyzed the data of the cases diagnosed as congenital hypothyroidism by neonatal screening program from January 1st 2003 to December 31th 2011 in Al Fayoum governorate to determine the prevalence of permanent and transient hypothyroidism in this period.

## Methodology

This descriptive retrospective study of the health insurance databases was conducted to determine the prevalence of permanent and transient hypothyroidism. Ethical committee of Faculty of Medicine, Fayoum University had approved the study. Consents were not taken from care givers as the study was done on health insurance databases with permission from health insurance center managerial board. The Public Health Insurance Organization is the governmental organization, responsible for newborn screening program. The Institution Review Board was aware of the public insurance system not taking consent to utilize data of patients and waived the need for consent. The Fayoum congenital hypothyroidism screening program began in January 2003 and is continuing.

All 3 to 7 day neonates were screened using dry blood spot on filter paper taken from a prick heel capillary blood sample. TSH was measured using enzyme linked immune-sorbent assay (ELISA). Samples were considered positive if the neonatal TSH (NTSH) concentration was ≥15μu/ml. If NTSH was ranging from 15 to 40μ u/ml another dry sample was taken within 2 days for re-measurement of NTSH. Samples were considered positive if the second NTSH was ≥15μu/ml and were sent for confirmatory test. Cases with a TSH level above 40μu/ml did not need another dry sample and were sent for confirmation test. The confirmatory test was performed in the central lab of ministry of health and population using venous blood samples obtained from the cubital vein on the day of referral and serum T4 and TSH levels were determined. The test results were compared to age related norms as presented in table (1) [12].

**Table 1:** Normal ranges for serum TSH & T4 in venous sample [12].

**Normal references for venous TSH**	
**Sample time**	**Normal value**
1-4 days	1-38.9 µu/L
2 weeks -2 years	0.6-9 µu/L
2 years -20 years	0.7-5.7 µu/L
	
**Normal references for venous T4**	
**Sample time**	**Normal value**
1-3 days	11.8-22.6ng/dl
1-2 weeks	8.9-16.6ng/dl
1-4 months	7.2-14.4ng/dl
4-12 months	7.8-16.5ng/dl
1-5 years	6.4-13.3ng/dl

Treatment was initiated for neonates whose TSH level was above 40μu/ml and those with the second dry spot sample level ≥15μu/ml without waiting for the confirmatory test results. If the results of the confirmatory test were within the normal limits the neonates were considered to have transient TSH elevation and treatment was halted. Neonates with confirmed congenital hypothyroidism were treated with levothyroxine 10–15µg/Kg/day, the dose may be increased or decreased based on normalization of T4/TSH laboratory check. Neonates with CH underwent thyroid scintigraphy with sodium pertechnetate to determine the etiology of hypothyroidism. Thyroid ultrasonography was performed when scintigraphy showed no uptake in the thyroid gland area to confirm thyroid agenesis.

Infant diagnosed with CH were followed every 2 weeks for the first 3 months, every month for the first year, every 2 months for the 2^nd^ and 3^rd^ years and every 6 months until adolescence is reached. Follow up visits to the health insurance centers during the first three years of life included anthropometric assessment, clinical examination, TSH/ total T4 levels, skeletal age determination using Greulich-Pyle standard, and neuro-developmental status using Bayley mental development scales.

Discontinuation of replacement treatment was tried at the age of three years in children suspected to have dyshormonogenesis based on imaging results, total daily dose ≤50µg/d, and normal clinical and laboratory follow up. Withdrawal of levothyroxine therapy was tried before the age of three years in children on total daily dose 12.5-25µg/day with normal clinical and laboratory evaluation. After one month of discontinuation of thyroxine treatment, T4/TSH levels were checked to ensure normal thyroid function without oral hormone substitution. These children were then repeatedly evaluated at regular intervals for one year to monitor thyroid function. Patients proved to have permanent hypothyroidism continued levothyroxine therapy and the initial dose was then adjusted according to the clinical and laboratory follow up.

Statistical analysis; Pre-coded data was entered on the computer using "Microsoft Office Excel Software" program (2010) for windows. Data was then transferred to the Statistical Package of Social Science Software program, version 20 (SPSS) to be statistically analyzed. Data was summarized using median and percentiles for quantitative variables and frequency and percentage for qualitative variables. Comparison between groups was done using Mann Whiney test for quantitative variables, chi square test with Fisher’s exact test for qualitative variables. P values equal to or less than 0.05 were considered statistically significant.

## Results

During the period from January 2003 to December 2011, 731743 neonates were screened.568 newborns were detected to have high TSH levels in the first and second dry samples. Twenty patients were lost to follow up and did not have their confirmatory test. The confirmatory test revealed normal T4 levels and mild elevation of TSH in 300 neonates. These newborns were considered to have transient elevation of TSH and followed up to insure normal thyroid function without replacement therapy. Of the screened newborns, 248 patients were diagnosed as hypothyroid and received treatment. Of these 248 patients, 161 (65%) patients had thyroid dysgenesis due to abnormal thyroid gland development involving various embryogenic defect{107 (66.5%) ectopic thyroid gland, 28 (17.4%) athyreosis and 26 (16.1%) thyroid hypoplasia}. 87 (35%) patients had intact gland in thyroid scan and were considered to have dyshormonogenesis. Of these 87 patients 44 (51%) proved to have transient hypothyroidism as clinical and laboratory follow up revealed normal thyroid profile after 1 month of discontinuation of treatment. The remaining 43 (49%) patients were diagnosed as permanent hypothyroidism as trial of withdrawal of replacement therapy was associated with reduction of T4 levels.

**Figure 3:**
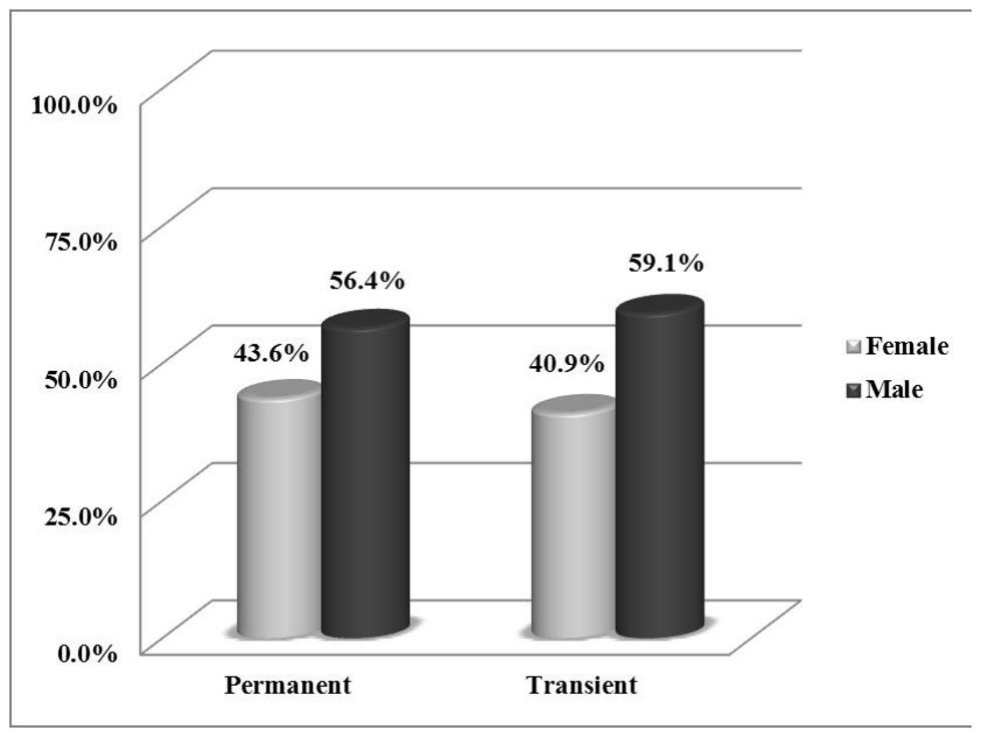
Bar chart showing the deference between Permanent and transient congenital hypothyroidism regarding sex.

**Figure 4:**
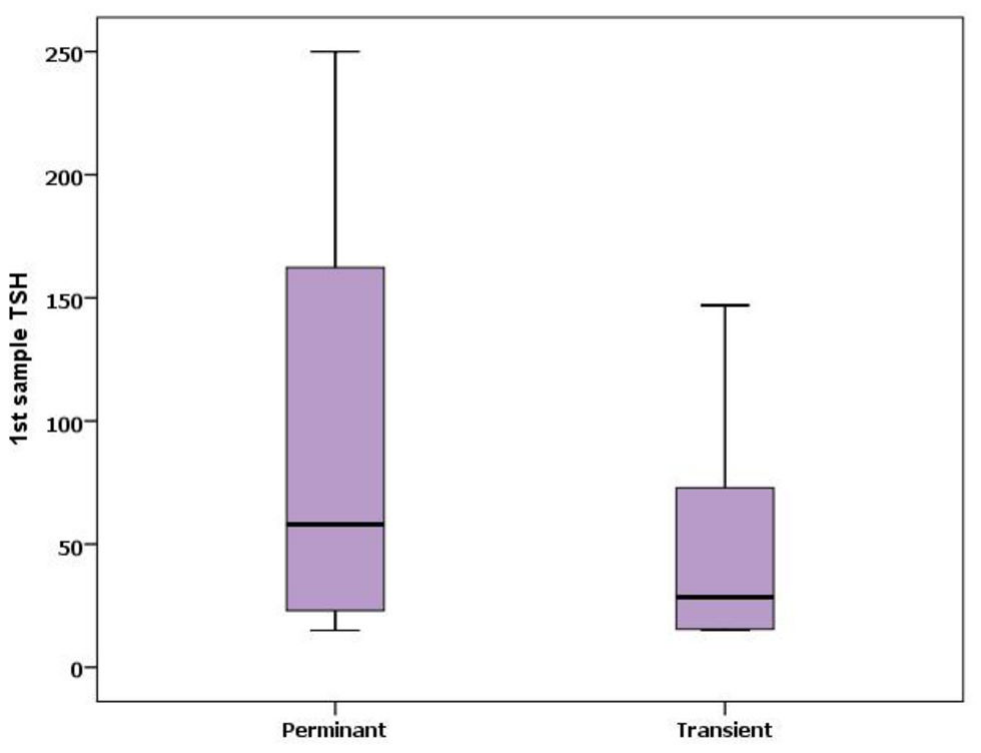
Box plot chart showing the differences between Permanent and transient congenital hypothyroidism regarding 1^st^ sample TSH.

The incidence of CH was 3.4/10000 live birth (1/2941 live birth). The female to male ratio was 0.8 (107/141). Of the 248 patients, 44 (17.7%) patients were diagnosed as transient hypothyroidism and 204 (82.3%) were diagnosed as permanent hypothyroidism {161 (79%) patients with thyroid dysgenesis and 43 (21%) with dyshormonogenesis}. The prevalence of permanent and transient CH was 1/3587 live birth, 1/16667 live birth respectively. The female to male ratio was 0.8 and 0.7 in permanent and transient CH respectively, as shown in Figure (3). The first sample TSH levels were significantly higher in permanent cases than transient cases (p< 0.004) as shown in Figure (4). Comparison of cases with permanent and transient hypothyroidism is presented in table (2).

**Table 2:** Characteristics of permanent & transient congenital hypothyroidism.

	**Permanent**		**Transient**		**P value**
**Sex** n, %					
Female	89	43.6	18	40.9	0.9
Male	115	56.4	26	59.1	
F / M ratio	89/115	0.8	18/26	0.7	
					
**1st sample TSH**	58 (23, 167.7)		28.5 (15.3, 73.4)		**0.004**
Median (25^th^, 75^th^ percentiles)					
**2^nd^ sample TSH**	73 (28, 157)		56.6 (26, 96.3)		0.08
Median (25^th^, 75^th^ percentiles)					
**Confirming TSH**	33.0 (18.3, 64.0)		15 (14, 44)		0.2
Median (25^th^, 75^th^ percentiles)					
**Confirming T4**	12.1 (8.9, 15.3)		6.6 (3.4, 23.3)		0.4
Median (25^th^, 75^th^ percentiles)					
**Age at start of treatment**	15 (12, 19)		15 (13, 17.8)		0.8
Median (25^th^, 75^th^ percentiles)					

Of the 44 children diagnosed with transient hypothyroidism, 39 (88.6%) children discontinued levothyroxine replacement therapy at the age of two years. These children received low dose levothyroxine; 30 (68.2%) children were given 12.5µg/d, whereas 9 (20.4%) were given initial dose 25µg/d which was reduced to 12.5µg/d during the first two years. Five (11.4%) children stopped treatment at the age of three years. These children received initial dose 50µg/d which was reduced to 25µg/d one month before trial of discontinuation.

Of the 204 patients diagnosed with permanent hypothyroidism, 4 patients died. The dose of levothyroxine in patients with permanent CH ranged from 50–125 µg/day with an average 73.5±27.2 (the median dose was 75 µg/day, with IQR 50–100). Of the remaining 200 patients, about three quarters 146 (73%) of cases were compliant to therapy while nearly one quarter 54 (27%) of cases were not. As regards the compliance to scheduled visits, most of patients 166 (83%) were always compliant and less than one fifth 34 (17%) were sometimes compliant. Among the cohort with good compliance to therapy 139 (95.2%) patients had normal height for age and 7 (4.8%) patients were less than 2 standard deviations (SD) height for age. Among the cohort with poor compliance to therapy only 33 (61.1%) patients had normal height for age and 21 (38.9%) patients height were less than 2 SD height for age associated with delayed bone age.

## Discussion

In this study incidence of CH was 1:2941 live birth. A similar incidence was reported in a study from the United States by Simpser and Rapaport that described the worldwide incidence of CH [13].

In our study 248 patients with primary hypothyroidism, 204 (82.3%) were diagnosed as permanent type and 44 (17.7%) were diagnosed as transient type. Studies from other parts of the world have reported incidence of transient hypothyroidism in 1–50% of children with congenital hypothyroidism [14–18]. It is generally accepted that 10–15% of primary CH patients are diagnosed with transient type [19]. In the study by Ordookhani et al of 35 neonates with primary CH, 25 (71.4%) had permanent CH, 6 (7%) had transient CH and 4 cases were unclassified [20]. In a study from Saudi Arabia only two of 24 neonates with CH had transient type [21]. The higher incidence of transient CH in our study may be owing to iodine deficiency in Egypt especially our governorate [11,22]; however urinary iodine excretion should be measured to confirm the diagnosis. In a study by Gaudino et al in France 38% and 62% of 79 patients with CH were reported to have transient and permanent CH respectively [23]. Incidence of transient CH was 28% in the United States as reported by Korzeniewski et al [24] and Mitchell et al [25]. Transient CH may be due to maternal factors such as iodine deficiency, excessive iodine intake, anti-thyroid medication or presence of antibodies against thyroid tissue during pregnancy [3,4,9]. Also very low birth weight (<1500gm), prematurity (<37 weeks gestation), immaturity of thyroidal iodine organification, exposure to excess iodine (e.g. use of iodinated disinfectants or contrast agents) and gene mutation may contribute to transient CH [5–7,8]. Children with transient CH show normal mental and physical development under thyroxine treatment. Treatment can be discontinued after two or three years [3,4].

In our study, prevalence of permanent CH was 1/3587 live birth. This is approximately equal to the worldwide incidence of CH [26]. Comparing the findings of our study with other international research suggests that incidence of permanent CH in Fayoum was nearly half the incidence in Greek 1/1200[27] and Saudi Arabia (1/1400) [28]. In a study from Isfahan – Iran the incidence of permanent CH was 4-5 times higher than the incidence in the present study [8]. These variations of incidences may be due to different environmental, genetic and immunological factors as well as ethnicity [29,30].

The female to male ratio was 0.8 and 0.7 in permanent and transient CH respectively. The female to male ratio of permanent hypothyroidism is similar to a study by Hashemipour et al [8]. Other studies reported a higher incidence among females compared with males [20,31]. This finding could be explained by the high parental consanguinity in our region and the undiagnosed family history of CH as reported also by Castanet et al [32].

The median TSH levels before treatment were significantly higher in patients with permanent CH than those with transient CH. Similar findings were reported by Hashemipour et al [8] in the study from Iran and by Nair et al in the study from India [33]. The median T4 levels before starting treatment were not significantly different among patients with permanent and transient CH. This was reported also by Hashemipour et al [8], while in other reports initial T4 levels correlated with the etiology of CH [34]. It was conducted from these findings that the first TSH and T4 levels may have predictive role for identifying permanent forms of CH from the transient forms.

The most common cause of permanent CH is thyroid dysgenesis [35]. In this study thyroid dysgenesis was the main cause of permanent CH accounting for 79% of cases and dyshormonogenesis accounts for the remaining 21%. These results were similar to other studies reported by Nair et al from india [33] and Ordookhani et al from Iran [20].

We concluded that the incidences of CH as well as the permanent form were similar to the worldwide reported ones. Although the high incidence of transient CH in our study could be explained by iodine deficiency further studies are needed to confirm the etiology and plan the treatment strategies.
